# Novel imidazopyridine suppresses STAT3 activation by targeting SHP-1

**DOI:** 10.1080/14756366.2018.1497019

**Published:** 2018-09-28

**Authors:** Jung-Chen Su, Chuan-Hsun Chang, Szu-Hsien Wu, Chung-Wai Shiau

**Affiliations:** a Institute of Biopharmaceutical Sciences, National Yang-Ming University, Taipei, Taiwan;; b Faculty of Pharmacy, National Yang-Ming University, Taipei, Taiwan;; c Chairman of the Surgical Department, Cheng Hsin General Hospital, Taipei, Taiwan;; d Department of Chemistry, Chung-Yuan Christian University, Chungli, Taiwan

**Keywords:** STAT3, SHP-1, HCC, imidazopyridine

## Abstract

The unregulated activation of STAT3 has been demonstrated to occur in many cancers and enhances tumour growth, migration, and invasion. Stimulation by cytokines, growth factors, and hormones triggers this activation by phosphorylating STAT3 at tyrosine 705. Novel imidazopyridine compounds were synthesized to evaluate the inhibition of STAT3 at Y705. Among the tested compounds, **16** reduced the level of phospho-STAT3, inhibited the downstream signalling cascade and subsequently attenuated the survival of hepatocellular carcinoma (HCC) cells. Further assays showed that the reduction effects of compound **16** on tyrosine 705 of STAT3 were attributed to up-regulation of protein tyrosine phosphatase SHP-1.

## Introduction

Recent cancer drug discovery has focused on molecular target therapy to reduce nonspecific cytotoxicity in patients. Targeting oncogenes or activating tumour suppressor genes with small molecules is a new approach for cancer treatment. Signal transducer and activator of transcription 3 (STAT3) is a transcription factor that plays a central role in tumour cell proliferation, survival, invasion, and immunosuppression^1^. The STAT3 pathway is activated by several receptors, including those for the interleukin-6 (IL-6) family cytokines^2^, G-protein-coupled receptors (GPCRs)^3^^,^^4^, and Toll-like receptors (TLRs)^5^. Once the signal is activated, STAT3 is phosphorylated at the tyrosine 705 residue, resulting in automatic dimerization in the cytosol. The dimeric STAT3 translocates from the cytosol to the nucleus for the transcription of its target genes, such as Mcl-1, Bcl-xl, and VEGF-A^6–8^ and further regulates cell proliferation, apoptosis, and migration. This phosphorylation activation of STAT3 is negatively regulated by protein tyrosine phosphatases, such as SHP-1, SHP-2, and PTP1B by targeting the phosphor group at tyrosine 705^9^^,^^10^. Therefore, the enhancement of protein tyrosine phosphatases is another approach for target therapy.

Potentiating the effect of traditional chemotherapy agents and targeting chemotherapy-induced signalling cascades is a practical approach for combating resistant cancer cells. In gastric cancer cells, inhibition of STAT3 phosphorylation re-sensitized the chemotherapeutic agent treatment^11^.

**Figure 1.**
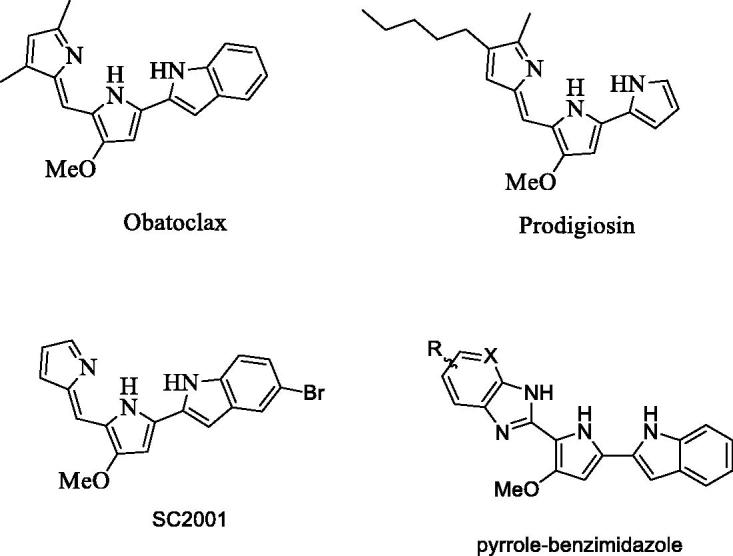
The structure of obatoclax, prodigiosin, SC-2001, and the core structure of pyrrole-benzimidazole.

Recently, not only prodigiosin, a natural product extracted from *Serratia marcescens*, but also obatoclax, a synthetic oligopyrrole scaffold derived from prodigiosin, have been shown to regulate biological and pharmacological effects (Figure 1)^12–14^. The combination of DNA damaging doxorubicin and prodigiosin showed significant growth inhibition in human small-cell lung doxorubicin-resistant cancer cells^15^. Obatoclax, currently in the clinical trial stage, has also been applied to synergize with targeted agents such as bortezomib^16^ and rituximab^17^ in various cancers, and also combined with Olaparib^18^ and Lapatinib^19^ in preclinical studies.

Previously, we designed and synthesized obatoclax derivatives by the replacement of the indole ring with thiophene and furan, resulting in a new chemical entity possibly suitable for the development of anticancer agents^20^. Moreover, these derivatives induced cell apoptosis through a novel mechanism by targeting the SHP-1/STAT3 pathway, a different mechanism from obatoclax to Mcl-1, Bcl-2, and Bcl-xL inhibition^21^. Therefore, our hypothesis is that pyrrole-benzimidazole could be a new pharmacophore for SHP-1 enhancement which represses STAT3 activation and induces cell apoptosis (Figure 1). In this study, we designed a short synthetic scheme for pyrrole-benzimidazole and synthesized various pyrrole-benzimidazole-based compounds aiming to find compounds that enhance SHP-1 activity (Scheme 1). The structure–activity relationship of these compounds was investigated using hepatocellular carcinoma (HCC) cancer cells. We further studied the SHP-1 activity and p-STAT-3 inhibition by treating HCC cells with the effective compounds.

## Materials and methods

### Chemistry

Proton nuclear magnetic resonance (^1^H-NMR and ^13^C-NMR) spectra were recorded on Bruker Avance III (400 MHz, Bruker, Billerica, MA) instruments. Reaction progress was determined by thin layer chromatography (TLC) analysis on a silica gel 60 F254 plate (Merck, Darmstadt, Germany). Chromatographic purification was carried out on silica gel columns 60 (0.063–0.200 mm or 0.040–0.063 mm; Merck), basic silica gel. Commercial reagents and solvents were purchased from Aldrich (Darmstadt, Germany) and Acros Chemical Corporation (Bridgewater, NJ) without additional purification. High-resolution mass spectra were recorded on a FINNIGAN MAT 95S mass spectrometer.

#### General procedure for the synthesis of compound ***5***

Compound **3** (1.0 equiv.) and NaHSO_3_ (2.0 equiv.) were mixed in ethanol solution. After the mixtures were heated with a 50 Watt microwave at 90 °C for 10 min, the solution was evaporated with rotavapor. The crude mixtures were added with a solution of dimethylformamide and 1.2 equiv benzene-diamine. The reaction was stirred in 50 Watt microwave reactor at 125 °C for 30 min, poured into 20 ml water and extracted with ethyl acetate (30 ml) 3 times. The organic layer was collected, washed with brine, dried with MgSO_4_. The concentrated crude product was purified by silica gel with elute (ratio: ethyl acetate/hexane =1/6 to 1/2). The yield of coupling product in this procedure was 4–24%.

### Tert-butyl-2-(5-(1H-benzo[d]imidazol-2-yl)-4-methoxy-1H-pyrrol-2-yl)-1H-indole-1-carboxylate (**6**)

*Tert*-butyl-2–(5-(1*H*-benzo[*d*]imidazol-2-yl)-4-methoxy-1*H*-pyrrol-2-yl)-1*H*-indol. ^1^H NMR (400 MHz, CDCl_3_): δ = 8.42 (s, 1H), 8.08 (d, *J* = 8.4 Hz, 1H), 7.52 (d, *J* = 7.2 Hz, 1H), 7.29 (t, *J* = 7.2 Hz, 1H), 7.04 (d, *J* = 7.6 Hz, 1H), 6.96 (t, *J* = 7.2 Hz, 1H), 6.83 (s, 1H), 6.73 (d, *J* = 7.2 Hz, 2H), 6.19 (s, 1H), 3.88 (s, 3H), 1.59 (s, 10H).

### 2–(5-(5-bromo-1H-indol-2-yl)-3-methoxy-1H-pyrrol-2-yl)-1H-benzo[d] imidazole (**7**)

^1^H NMR (400 MHz, MeOH-d_4_): δ = 7.63 (d, *J* = 1.6 Hz, 1H), 7.52 (dd, *J* = 6.0, 3.2 Hz, 2H), 7.25 (d, *J* = 8.4 Hz, 1H), 7.1 5–7.18 (m, 3H), 6.67 (s, 1H), 6.49 (s, 1H), 4.02 (s, 3H) ppm. ^13 ^C NMR (100 MHz, MeOH-d_4_): δ = 151.4, 146.5, 139.5, 136.8, 133.3, 131.9, 127.1, 125.1, 123.0, 122.8, 115.3, 115.1, 114.8, 114.1, 113.5, 113.1, 108.2, 97.6, 94.3, 58.5 ppm.

### 2–(5-(1H-indol-2-yl)-3-methoxy-1H-pyrrol-2-yl)-1H-benzo[d]imidazole (**8**)

^1^H NMR (400 MHz, MeOH-d_4_): δ = 7.5 1–7.54 (m, 3H), 7.36 (d, *J* = 8.2 Hz, 1H), 7.1 7–7.19 (m, 2H), 7.10 (t, *J* = 8.2 Hz, 1H), 7.01 (t, *J* = 7.6 Hz, 1H), 6.73 (s, 1H), 6.51 (s, 1H), 4.05 (s, 3H) ppm. ^13 ^C NMR (100 MHz, MeOH-d_4_): δ = 151.9, 146.8, 138.4, 132.0, 130.3, 128.2, 123.1, 122.8, 121.0, 120.7, 114.9, 111.8, 107.8, 98.4, 94.1, 58.7 ppm. HRMS calculated for C_20_H_16_N_4_O (M + H): 329.1402, Found: 329.1403.

### 2–(4-methoxy-1H, 1'H-[2, 2'-bipyrrol]-5-yl)-1H-benzo[d]imidazole (**9**)

^1^H NMR (400 MHz, MeOH-d_4_): δ = 7.4 7 ∼ 7.49 (m, 2H), 7.1 3 ∼ 7.15 (m, 2H), 6.78 (dd, *J* = 3.2, 1.6 Hz, 1H), 6.42 (dd, *J* = 3.2, 1.6 Hz, 1H), 6.23 (s, 1H), 6.16 (t, *J* = 3.2 Hz, 1H), 4.01 (s, 3H) ppm. HRMS calculated for C_16_H_14_N_4_O (M + H): 279.1246, Found: 279.1248.

### 6-bromo-2-(5-(5-bromo-1H-indol-2-yl)-3-methoxy-1H-pyrrol-2-yl)-3H-imidazo[4,5-b]pyridine (**10**)

^1^H NMR (400 MHz, MeOH-d_4_): δ = 8.30 (s, 1H), 7.98 (s, 1H), 7.67 (d, *J* = 2.0 Hz, 1H), 7.30 (d, *J* = 8.8 Hz, 1H), 7.20 (dd, *J* = 8.8, 2.0 Hz, 1H), 6.77 (s, 1H), 6.54 (s, 1H), 4.07 (s, 3H) ppm. ^13 ^C NMR (100 MHz, MeOH-d_4_): δ = 136.8, 132.8, 131.8, 129.0, 125.4, 123.1, 113.5, 113.2, 98.4, 96.7, 94.2, 58.5 ppm.

### 2–(5-(5-bromo-1H-indol-2-yl)-3-methoxy-1H-pyrrol-2-yl)-3H-imidazo[4,5-b]pyridine (11)

^1^H NMR (400 MHz, MeOH-d_4_): δ = 8.24 (d, *J* = 3.6 Hz, 1H), 7.85 (d, *J* = 8.0 Hz, 1H), 7.65 (s, 1H), 7.26 (d, *J* = 8.8 Hz, 1H), 7.1 6–7.19 (m, 2H), 6.72 (s, 1H), 6.50 (s, 1H), 4.07 (s, 1H) ppm. ^13 ^C NMR (100 MHz, MeOH-d_4_): δ = 153.1, 143.7, 137.1, 133.2, 132.1, 128.7, 125.6, 123.4, 120.1, 118.3, 113.8, 113.4, 107.9, 98.4, 94.4, 58.8 ppm. HRMS calculated for C_19_H_14_BrN_5_O (M + H): 408.0460, Found: 408.0459.

### 2–(5-(5-bromo-1H-indol-2-yl)-3-methoxy-1H-pyrrol-2-yl)-5-fluoro-1H-benzo[d]imidazole (12)

^1^H NMR (400 MHz, MeOH-d_4_): δ = 7.60 (d, *J* = 1.6 Hz, 1H), 7.43 (dd, *J* = 8.8, 4.8 Hz, 1H), 7.22 (d, *J* = 8.8 Hz, 1H), 7.19 (dd, *J* = 9.2, 2.4 Hz, 1H), 7.14 (dd, *J* = 8.8, 1.6 Hz, 1H), 6.91 (td, *J* = 9.2, 2.4 Hz, 1H), 6.63 (s, 1H), 6.43 (s, 1H), 3.98 (s, 3H) ppm. ^13 ^C NMR (100 MHz, MeOH-d_4_): δ = 161.9, 159.5, 151.8, 137.0, 133.5, 133.4, 132.1, 132.0, 127.5, 125.4, 123.2, 113.7, 113.3, 110.7, 110.5, 108.3, 97.9, 94.5, 58.7 ppm. HRMS calculated for C_20_H_14_BrFN_4_O (M–H): 423.0257, Found: 423.0243.

### 2–(5-(5-bromo-1H-indol-2-yl)-3-methoxy-1H-pyrrol-2-yl)-1H-benzo[d] imidazole-5-carbonitrile (13)

^1^H NMR (400 MHz, MeOH-d_4_): δ = 7.86 (s, 1H), 7.66 (d, *J* = 2.0 Hz, 1H), 7.64 (d, *J* = 8.0 Hz, 1H), 7.49 (d, *J* = 8.0 Hz, 1H), 7.28 (d, *J* = 8.4 Hz, 1H), 7.19 (dd, *J* = 8.4, 2.0 Hz, 1H), 6.75 (s, 1H), 6.54 (s, 1H), 4.07 (s, 3H) ppm. HRMS calculated for C_21_H_14_BrN_5_O (M-H): 430.0303, Found: 430.0326

### 3–((2–(5-(5-bromo-1H-indol-2-yl)-3-methoxy-1H-pyrrol-2-yl)-1H-benzo[d] imidazol-6-yl)oxy)aniline (14)

^1^H NMR (400 MHz, MeOH-d_4_): δ = 7.65 (d, *J* = 1.6 Hz, 1H), 7.48 (d, *J* = 8.8 Hz, 1H), 7.27 (d, *J* = 8.4 Hz, 1H), 7.18 (dd, *J* = 8.4, 1.6 Hz, 1H), 7.15 (d, *J* = 2.0 Hz, 1H), 7.03 (t, *J* = 8.0 Hz, 1H), 6.90 (dd, *J* = 8.8, 2.0 Hz, 1H), 6.69 (s, 1H), 6.52 (s, 1H), 6.43 (dd, *J* = 8.0, 2.0 Hz, 1H), 6.36 (t, *J* = 2.4 Hz, 1H), 6.30 (dd, *J* = 8.0, 2.4 Hz, 1H), 4.04 (s, 1H) ppm.

### 3–((2–(5-(5-bromo-1H-indol-2-yl)-3-methoxy-1H-pyrrol-2-yl)-1H-benzo[d] imidazol-6-yl)oxy)benzonitrile (15)

^1^H NMR (400 MHz, MeOH-d_4_): δ = 7.65 (d, *J* = 2 Hz, 1H), 7.57 (d, *J* = 8.4 Hz, 1H), 7.49 (t, *J* = 7.6 Hz, 1H), 7.40 (d, *J* = 7.6 Hz, 1H), 7.2 6 ∼ 7.28 (m, 3H), 7.24 (d, *J* = 2.4 Hz, 1H), 7.18 (dd, *J* = 8.8, 2 Hz, 1H), 6.94 (dd, *J* = 8.4, 2.4 Hz, 1H), 6.71 (s, 1H), 6.53 (s, 1H), 4.05 (s, 3H) ppm. ^13 ^C NMR (100 MHz, MeOH-d_4_): δ = 152.2, 151.9, 137.0, 133.5, 132.1, 127.0, 125.4, 123.3, 123.1, 121.3, 120.5, 119.2, 116.0, 114.5, 113.7, 113.4, 98.0, 94.6, 58.8 ppm.

### 2–(5-(1H-indol-2-yl)-3-methoxy-1H-pyrrol-2-yl)-6-bromo-3H-imidazo[4,5-b]pyridine (16)

^1^H NMR (400 MHz, MeOH-d_4_): δ = 8.25 (s, 1H), 7.91 (s, 1H), 7.51 (d, *J* = 8.0 Hz, 1H), 7.34 (d, *J* = 8.0 Hz, 1H), 7.10 (t, *J* = 8.0 Hz, 1H), 7.02 (t, *J* = 8.0 Hz, 1H), 6.76 (s, 1H), 6.47 (s, 1H), 4.04 (s, 3H) ppm. ^13 ^C NMR (100 MHz, MeOH-d_4_): δ = 153.7, 144.2, 138.5, 131.6, 130.3, 130.1, 123.1, 121.2, 120.8, 111.9, 107.2, 99.3, 94.0, 58.6 ppm. HRMS calculated for C_19_H_14_BrN_5_O (M-H): 406.0303, Found: 406.0302.

### 2–(5-(1H-indol-2-yl)-3-methoxy-1H-pyrrol-2-yl)-3H-imidazo[4,5-b]pyridine (17)

^1^H NMR (400 MHz, MeOH-d_4_): δ = 8.23 (d, *J* = 4.4 Hz, 1H), 7.84 (d, *J* = 8.0 Hz, 1H), 7.53 (d, *J* = 7.6 Hz, 1H), 7.36 (d, *J* = 8.0 Hz, 1H), 7.17 (dd, *J* = 8.0, 4.8 Hz, 1H), 7.11 (t, *J* = 7.6 Hz, 1H), 7.01 (t, *J* = 7.6 Hz, 1H), 6.77 (s, 1H), 6.50 (s, 1H), 4.06 (s, 3H) ppm. ^13 ^C NMR (100 MHz, MeOH-d_4_): δ = 153.2, 143.5, 138.5, 131.7, 130.3, 129.5, 122.9, 121.1, 120.7, 118.2, 111.8, 107.4, 98.9, 93.9, 58.7 ppm. HRMS calculated for C_19_H_15_N_5_O (M + H): 330.1355, Found: 330.1344.

### 2–(5-(1H-indol-2-yl)-3-methoxy-1H-pyrrol-2-yl)-5-fluoro-1H-benzo[d] imidazole (18)

^1^H NMR (400 MHz, MeOH-d_4_): δ = 7.50 (d, *J* = 8.0 Hz, 1H), 7.43 (dd, *J* = 8.4, 4.8 Hz, 1H), 7.34 (d, *J* = 8.0 Hz, 1H), 7.20 (dd, *J* = 9.2, 2.4 Hz, 1H), 7.08 (td, *J* = 7.6, 1.2 Hz, 1H), 6.99 (td, *J* = 7.6, 1.2 Hz, 1H), 6.91 (td, *J* = 9.2, 2.4 Hz, 1H), 6.70 (s, 1H), 6.45 (s, 1H), 4.00 (s, 3H) ppm. ^13 ^C NMR (100 MHz, MeOD-d_4_): δ = 161.9, 159.5, 152.1, 138.4, 131.9, 130.3, 128.4, 122.8, 121.0, 120.7, 111.8, 110.7, 110.5, 98.6, 94.0, 58.7 ppm. HRMS calculated for C_20_H_15_FN_4_O (M–H): 345.1152, Found: 345.1143.

### 3–((2-(5-(1H-indol-2-yl)-3-methoxy-1H-pyrrol-2-yl)-1H-benzo[d]imidazol-6-yl)oxy)aniline (19)

^1^H NMR (400 MHz, MeOH-d_4_): δ = 7.52 (d, *J* = 7.6 Hz, 1H), 7.41 (d, *J* = 8.4 Hz, 1H), 7.36 (d, *J* = 8.0 Hz, 1H), 7.15 (d, *J* = 2.4 Hz, 1H), 7.10 (t, *J* = 7.2 Hz, 1H), 6.9 9–7.07 (m, 2H), 6.90 (dd, *J* = 8.4, 2.4 Hz, 1H), 6.73 (s, 1H), 6.51 (s, 1H), 6.43 (dd, *J* = 8.0, 2.0 Hz, 1H), 6.36 (t, *J* = 2.4 Hz, 1H), 6.30 (dd, *J* = 8.0, 2.4 Hz, 1H), 4.05 (s, 3H) ppm.

### 6–bromo-2-(4-methoxy-1H, 1'H-[2, 2'-bipyrrol]-5-yl)-3H-imidazo[4,5-b] pyridine (20)

^1^H NMR (400 MHz, MeOH-d_4_): δ = 8.24 (s, 1H), 7.92 (d, *J* = 2.0 Hz, 1H), 6.82 (dd, *J* = 2.8, 1.2 Hz, 1H), 6.50 (dd, *J* = 3.6, 1.2 Hz, 1H), 6.26 (s, 1H), 6.18 (dd, *J* = 3.6, 2.8 Hz, 1H), 4.03 (s, 3H) ppm. ^13 ^C NMR (100 MHz, MeOH-d_4_): δ = 154.2, 146.3, 131.6, 129.1, 125.7, 120.1, 112.9, 110.1, 106.8, 106.5, 105.4, 91.8, 89.8, 81.5, 58.7 ppm. HRMS calculated for C_15_H_12_BrN_5_O (M–H): 356.0147, Found: 356.0152.

### 2–(4-methoxy-1H, 1'H-[2, 2'-bipyrrol]-5-yl)-1H-benzo[d]imidazole-5-carbonitrile (21)

^1^H NMR (400 MHz, MeOH-d_4_): δ = 7.80 (s, 1H), 7.58 (d, *J* = 8.4 Hz, 1H), 7.45 (dd, *J* = 8.4, 1.6 Hz, 1H), 6.81 (dd, *J* = 3.2, 1.6 Hz, 1H), 6.47 (dd, *J* = 3.2, 1.6 Hz, 1H), 6.25 (s, 1H), 6.17 (t, *J* = 3.2 Hz, 1H), 4.03 (s, 3H) ppm.

### (E)-N^4^-((5–(5-bromo-1H-indol-2-yl)-3-methoxy-1H-pyrrol-2-yl)methylene) pyrimidine-4,5-diamine (22)

Compound **3** (1.0 equiv.), pyrimidine-4,5-diamine (1.2 equiv) and NaHSO_3_ (2.0 equiv.) were mixed in the solution of ethanol. After the mixtures were heated with a 50 Watt microwave at 100 °C for 30 min, the solution was evaporated with rotavapor. The crude mixtures were poured into 20 ml water and extracted with ethyl acetate (30 ml) 3 times. The organic layer was collected, washed with brine, dried with MgSO_4_. The concentrated crude product was purified by silica gel with elute (ratio:ethyl acetate/hexane =1/1). Yield: 8.2%. ^1^H NMR (400 MHz, MeOH-d_4_): δ = 8.44 (d, *J* = 1.6 Hz, 1H), 8.17 (d, *J* = 1.6 Hz, 1H), 7.95 (d, *J* = 1.6 Hz, 1H), 7.67 (s, 1H), 7.29 (d, *J* = 8.4 Hz, 1H), 7.21 (d, *J* = 8.8 Hz, 1H), 6.86 (s, 1H), 6.45 (d, *J* = 1.6 Hz, 1H), 3.94 (s, 3H) ppm.

### (Z)-3-(2-((1H-pyrrol-2-yl)methylene)-3-methoxy-2H-pyrrol-5-yl)pyridine (23)

Pd(PP_3_)_4_ (0.1 equiv) was added to a solution of 1.0 equiv (*Z*)-2-((1*H*-pyrrol-2-yl)methylene)-3-methoxy-2*H*-pyrrol-5-yl trifluoromethanesulfonate (0.086 g, 0.267 mmole), Na_2_CO_3_ (0.028 g, 0.264 mmole), and 1.2 equiv pyridin-3-ylboronic acid (0.039 g, 0.317 mmole) in 10% water/dioxane (5 ml) purged with nitrogen. After the mixtures were heated at 100 °C for 90 min, the reaction was quenched with water. The mixture was extracted with ethyl acetate (30 ml) 3 times. The organic layer was collected, washed with brine, and dried with MgSO_4_. The concentrated crude product was purified by silica gel with elute (ratio: ethyl acetate/hexane =1/1). Yield: 35.6%. ^1^H NMR (400 MHz, CDCl_3_): δ = 9.18 (d, *J* = 1.6 Hz, 1H), 8.61 (dd, *J* = 4.8, 1.6 Hz, 1H), 8.26 (d, *J* = 8.0 Hz, 1H), 7.36 (dd, *J* = 8.0, 4.8 Hz, 1H), 7.24 (s, 1H), 7.18 (s, 1H), 7.01 (s, 1H), 6.68 (d, *J* = 3.6 Hz, 1H), 6.29 (dd, *J* = 3.6, 2.8 Hz, 1H), 6.05 (s, 1H), 3.91 (s, 3H) ppm.

## Biological assays

### Cell culture

Hepatoma cells PLC/PRF/5 (PLC5) were purchased from ATCC (Manassas, VA) (CRL-8024) and maintained in DMEM supplemented with 10% FBS, 100 units/mL penicillin G, 100 mg/mL streptomycin sulphate and 25 mg/mL amphotericin B in a 37 °C humidified incubator in an atmosphere of 5% CO_2_ in air.

### Cell viability assay

The effect of individual test agents on cell viability was assessed by using the 3-(4,5-dimethylthiazol-2-yl)-2,5-diphenyltetrazolium bromide (MTT) assay in five replicates. Cells were seeded and incubated in 96-well, flat-bottomed plates for 24 h and were exposed to various concentrations (2.5, 5, 10, 20, 40 μM) of test agents dissolved in DMSO (final concentration, 0.1%) in media. Controls received DMSO vehicle at a concentration equal to that in drug-treated cells. For the MTT assay, 10 μL of MTT dye was directly added to the wells. After 2 h, media were removed and then the cells were lysed with 100 μL of DMSO. The absorbance at 570 nm and 630 nm (background) were read with a microplate reader.

### Western blot

Lysates of PLC5 treated with compounds at the indicated concentrations for 24 h were analysed by western blot. p-STAT3 (#9145, 1:1000), STAT3 (#4904, 1:1000), Mcl-1 (#4572, 1:1000), survivin (#2803, 1:1000), JAK2 (#3230, 1:1000), GAPDH (#8884, 1:5000), and actin (#4967, 1:5000) antibodies were purchased from Cell Signaling (Danvers, MA). SHP-1 (ab32559, 1:1000) and cyclin D1 (ab24249, 1:1000) antibodies were purchased from Abcam (Cambridge, MA). PTP1B (2066–1, 1:2000) antibody was purchased from Epitomics (Cambridge, MA). p-JAK2(Tyr1007/1008) (44–42 G, 1:1000) antibody was purchased from ThermoFisher Scientific (Waltham, MA).

### SHP-1 phosphatase activity

A RediPlate 96 EnzChek Tyrosine Phosphatase Assay Kit (R-22067) was used for SHP-1 activity assay (Molecular Probes, Carlsbad, CA). The method was as described previously^22^.

### STAT3 activity assay

PLC5 cells were treated with the indicated compounds in 10 μM for 24 h and then analysed by the PathScan Phospho-Stat3 (Tyr705) Sandwich ELISA Kit (Cell Signaling)

### Gene knockdown using siRNA

Smart-pool small interfering RNAs (siRNAs), including the control (D-001810–10), SHP-1, were purchased from Dharmacon (Chicago, IL). The knockdown procedure was as described previously^23^.

### Statistical analysis

Data are expressed as mean ± SD. All statistical analyses were performed using SPSS for Windows version 12.0 software (SPSS Inc, Chicago, IL).

## Results and discussion

### Chemistry

The synthesis of the pyrrole-benzimidazole-based compounds was initiated with 4-methoxy-1H-pyrrol-2-one and dimethyl amide in the presence of phosphoryl tribromide to generate two functional groups, enamine, and bromine in the pyrrole ring **2**. The position of the brominated pyrrole ring was soon conjugated with aromatic boron acid in the presence of Pd(PPh_3_)_4_ by using the Suzuki coupling reaction and enamine was hydrolysed to the carbonyl group in acid conditions, resulting in the key intermediate **3**. The condensation reaction of the carbonyl group of **3** and various aromatic diamines under the different conditions resulted in the final imine **4** or pyrrole-imidazopyridine **5** products.

### Biological evaluation

All the new pyrrole-benzimidazole derivatives were analysed by MTT assay for growth inhibition against HCC cancer cells. The various substituents attached to the scaffold are summarized in Tables 1–5. The growth inhibition of each of the compounds is shown with an IC_50_ value which was calculated by interpolation from the dose–response curve in MTT assay.

**Table 1.** Chemical structure of compounds **6**–**9** and IC_50_ of cell death in PLC5 cells.

**Table 2.** Chemical structure of compounds **7**, and **10**–**15** and IC_50_ of cell death in PLC5 cells.

**Table 3.** Chemical structure of compounds **8**, and **16**–**19** and IC_50_ of cell death in PLC5 cells.

**Table 4.** Chemical structure of compounds **9**, **20**, and **21** and IC_50_ of cell death in PLC5 cells.

**Table 5.** Chemical structure of compounds **9**, **11**, **16**, **17**, **22**, and **23** and IC_50_ of cell death in PLC5 cells.

A set of pyrrole-benzimidazole derivatives was generated in which an indole, bromoindole, pyrrole, and Boc-indole connected to the methoxyl-pyrrole ring in the centre of the core moiety. *In vitro* test of cell death activity with these compounds against PLC5 cells showed differentiated activity with various substituents. Among these substituents, the indole ring **8** exhibited slightly better activity than bromo-indole **7** and pyrrole **9** (Table 1). In addition, the Boc-indole substituent **6** showed no activity against cell growth compared with the indole ring, suggesting the nitrogen atom plays an important role in the potency of cell growth. We next tested our hypothesis that the presence of various functional groups on benzimidazole might exhibit anti-cancer cell growth effects. A series of substituents, such as bromo, fluoro, cyano, and phenyl-cyano groups were thus introduced to the benzene and pyridine ring of benzimidazole to obtain a structure–activity relationship. These agents were tested in an MTT assay with PLC5 cells and the inhibition results are shown in Table 2. The pyridine ring of compound **11** demonstrated better activity than the benzene ring. In addition, Compound **13** with a cyano group connected to the benzene ring led to a reduction in inhibition of cell growth. Interestingly, the introduction of aniline **14** or phenyl-cyano **15** to benzimidazole resulted in no activity against PLC5 cells, implying that the linear electron-withdrawing group and phenyl substituent impede the anticancer activity. Analogues **17**–**19** that contained indole and imidazopyridine on both sides of pyrrole were also assayed for the inhibition of cell growth. As shown in Table 3, compounds **16** and **17** showed equal potency to compound **11**. Compound **20**, with imidazopyridine as a substituent to replace benzimidazole, led to a significant increase in anticancer activity. (Tables 4 and 5).

#### Mechanistic study of pyrrole-imidazopyridine in PLC 5 cells

**Figure 2.**
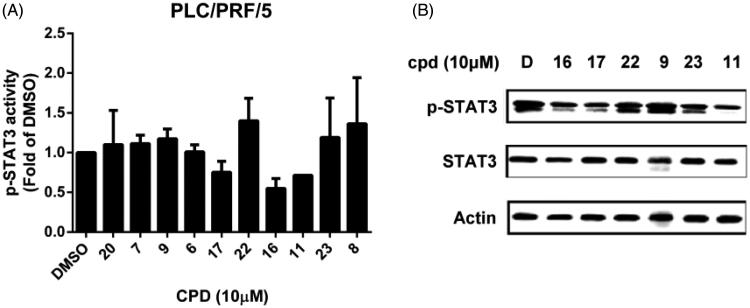
Effects of compounds on p-STAT3 inhibition. (A) PLC5 cells were exposed to the indicated compounds at a dose of 10 μM for 24 h and cell lysates were assayed by p-STAT3 ELISA kit. (B) PLC5 cells were exposed to the indicated compounds (cpd 16, 17, 22, 9, 23, 11) at 10 μM for 24 h and cell lysates were assayed by western blot.

**Figure 3.**
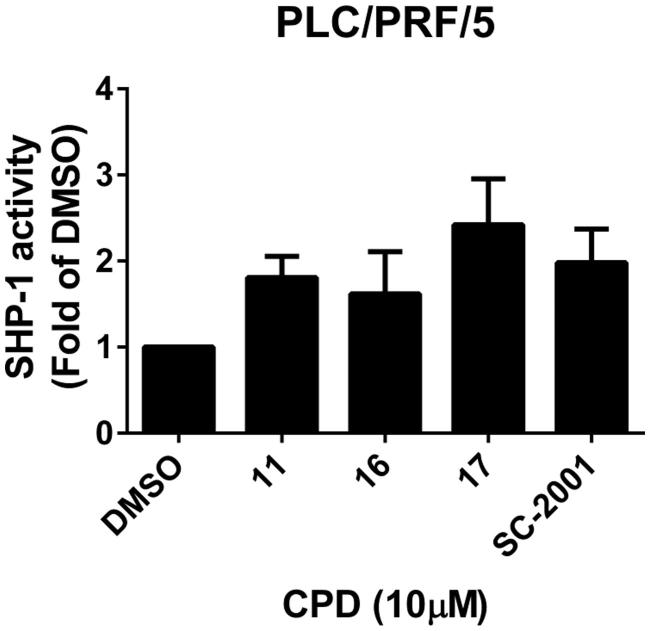
Effects of compounds on SHP-1 activity. PLC5 cells were exposed to cpd 11, 16, 17, and SC-2001 at 10 μM for 24 h and cell lysates were assayed by SHP-1 phosphatase activity kit.

**Figure 4.**
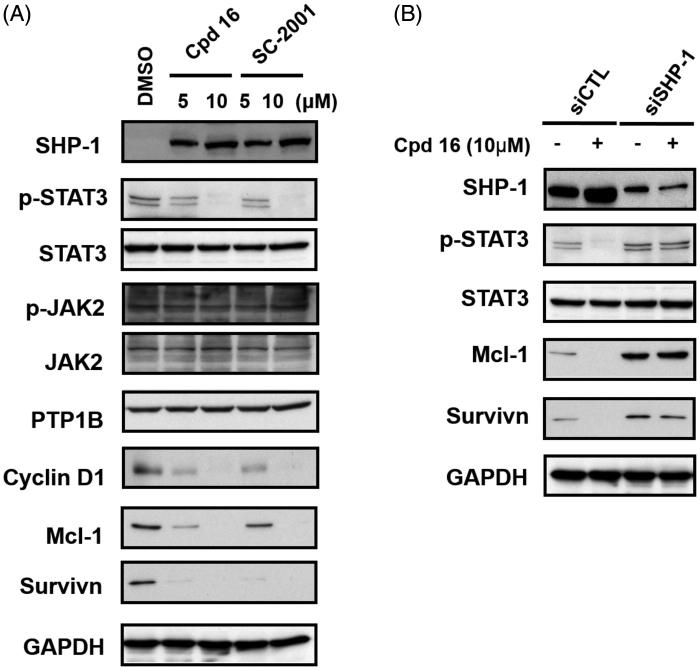
Effects of cpd 16 on protein levels of SHP-1, p-STAT3, and p-STAT3 relative downstream target in PLC5 cells. (A) Cells were exposed to cpd 16 and SC-2001 at the indicated doses for 24 h. Cell lysates were assayed by western blot. (B) PLC5 cells were transfected, respectively, with control siRNA or SHP-1 siRNA for 48 h. After transfection, the cells were treated w/wo cpd 16 (10 μM) for 24 h. The protein levels were analysed by western blot assay.

Previously, we have shown that indole-pyrrole reduces STAT3 phosphorylation in western blot assay and further represses cancer cell growth^20^. To further study whether the newly synthesized compounds had STAT3 inhibition activity, we screened these compounds using a p-STAT3 ELISA kit. Compounds **11**, **16,** and **17** significantly repressed STAT3 activity on PLC 5 cells (Figure 2(A)). These results correlated with the potency of cell growth inhibition. We further analysed 6 compounds to PLC5 cells with western blot assay and studied the relationship of cell growth and the status of STAT3. As shown in Figure 2(B), compounds **11**, **16**, and **17** resulted in a significant reduction of p-STAT3. In contrast, compounds **9**, **22,** and **23,** which were showed no cell growth inhibition, showed no appreciable change in inhibition of STAT3 as compared with the control panel. We further examined SHP-1 activity, the negative regulator of STAT3, by analysing the dephosphorylating effect in PLC5 cells. Compounds **11**, **16**, and **17** increased SHP-1 activity two-fold compared with the control panel (Figure 3). Meanwhile, we also explored the downstream signal cascade induced by compound **16** and **SC-2001** in PLC5 cells. The downstream targets of STAT3, such as cyclin D1, survivin, and Mcl-1, were decreased in the treatment of compound **16** and **SC-2001**, indicating that SHP-1 phosphatase activation and further p-STAT3 reduction were the major effectors in regulating cell survival. In addition, both of compound **16** and **SC-2001** had no effects on PTP-1B, p-JAK2, and JAK2 expressions (Figure 4(A)). Moreover, silencing SHP-1 with small-interference RNA (siRNA) abolished the effects of compound **16** on STAT3 de-phosphorylation and down-regulation of Mcl-1 and survivin (Figure 4(B)).

**Figure:**
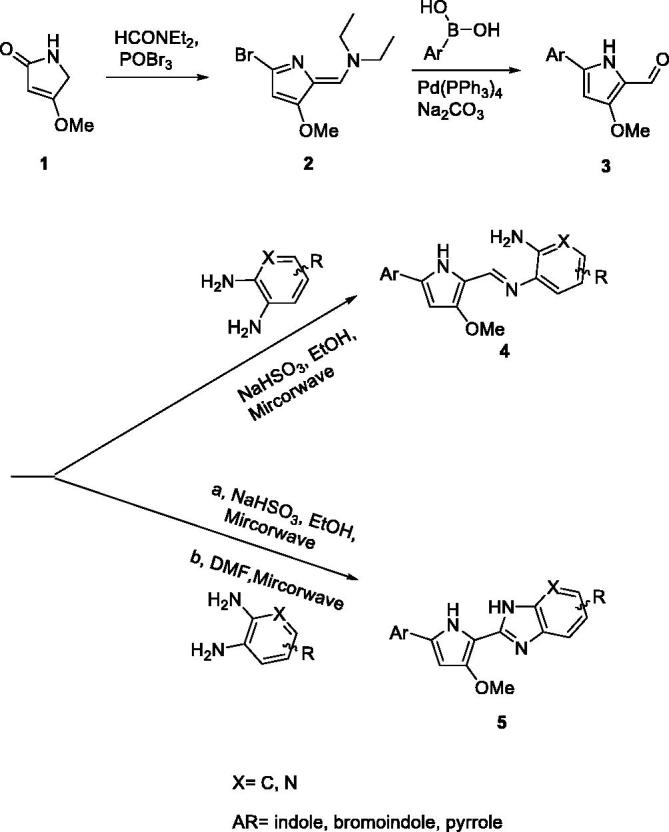
**Scheme** 1. General synthetic procedure for pyrrole-benzimidazole.

## Discussion

In this study, we studied the pharmacological effect of imidazopyridine derivatives and explored compound-induced blockade of cell signals which regulated HCC cell growth. We first designed an efficient synthetic route for the imidazopyridine core structure and derived a series of analogues for the anticancer test in PLC5 cells. These synthetic routes simplify the complicated procedure of amplifying a large amount of imidazopyridine and imine-pyridine for future *in vivo* studies by using commercially available starting materials and reagents. Based on the structure–activity relationship for cell growth inhibition, imidazopyridine substituents connected to an indole–pyrrole core are crucial. Replacement of imidazopyridine with imine-pyridine led to the loss of activity.

SHP-1 is reported to be a phosphatase in hematopoietic cells and executed de-phosphorylation activity to its target proteins in some biological processes. STAT3 is a major target of SHP-1 and highly expressed in different cancer cell types, including HCC^24^^,^^25^. Some reports have indicated that small molecules can negatively regulate cell growth through activating SHP-1 and further eliminating the phosphor group from active p-STAT3^26–28^. These phosphatase agonists provide a new direction for future clinical approaches instead of only kinase inhibition. Our discovery also provides evidence that imidazopyridine derivatives mediate both SHP-1 activation and also increase SHP-1 expression. Importantly, the reduction of cell survival genes, such as cyclin D1, survivin, and Mcl-1 regulated by SHP-1 were consistent with cell survival in the treatment of imidazopyridine derivatives.

In summary, this study showed that the structure of indole–pyrrole-imidazopyridine is a novel anticancer agent. We also demonstrated that these compounds can enhance SHP-1 expression and activation and consequently reduced the level of p-STAT3 in HCC cells. This indicates that the strategy of SHP-1 activation is a possible drug target in cancer patients who have no or low SHP-1 activity. In addition, these agents might be applied along with therapeutic cancer drugs to increase efficacy and reduce toxicity.
